# Potential of ITO thin film for electrical probe memory applications

**DOI:** 10.1080/14686996.2018.1534072

**Published:** 2018-10-15

**Authors:** Lei Wang, Jing Wen, Cihui Yang, Bangshu Xiong

**Affiliations:** School of Information Engineering, Nanchang Hangkong University, Nanchang, P. R. China

**Keywords:** ITO, electrical probe, phase-change, modeling, optimization, 40 Optical, magnetic and electronic device materials, 201 Electronics / Semiconductor / TCOs

## Abstract

Electrical probe memory has received considerable attention during the last decade due to its prospective potential for the future mass storage device. However, the electrical probe device with conventional diamond-like carbon capping and bottom layers encounters with large interfacial contact resistance and difficulty to match the experimentally measured properties, while its analog with titanium nitride capping and bottom layers also faces serious heat dissipation through either probe and silicon substrate. Therefore, the feasibility of using indium tin oxide (ITO) media for the capping and bottom layers of the electrical probe device is investigated by tailoring the thickness and electrothermal properties of the ITO capping and bottom layers within experimentally established range and subsequently calculating the resultant temperature at several predefined points based on a previously developed three-dimensional model. To meet the required temperature and to fit the experimentally reported values, the thickness, electrical conductivity, and thermal conductivity of the ITO capping and bottom layers are found to be 5 nm, 10^3^ Ω^−1^ m^−1^, 0.84 W m^−1^ K^−1^, and 200 nm, 1.25 × 10^6^ Ω^−1^ m^−1^, 0.84 W m^−1^ K^−1^, respectively. The practicality of using this optimized device to achieve ultrahigh density, ultralow energy consumption, ultrafast switching speed, low interfacial contact resistance, and high thermal reliability has also been demonstrated.

## Graphical Abstract

**Figure:**
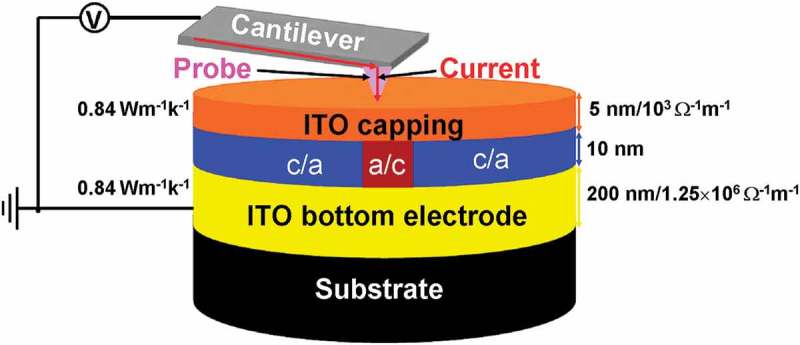

## 1. Introduction

**Figure 1.**
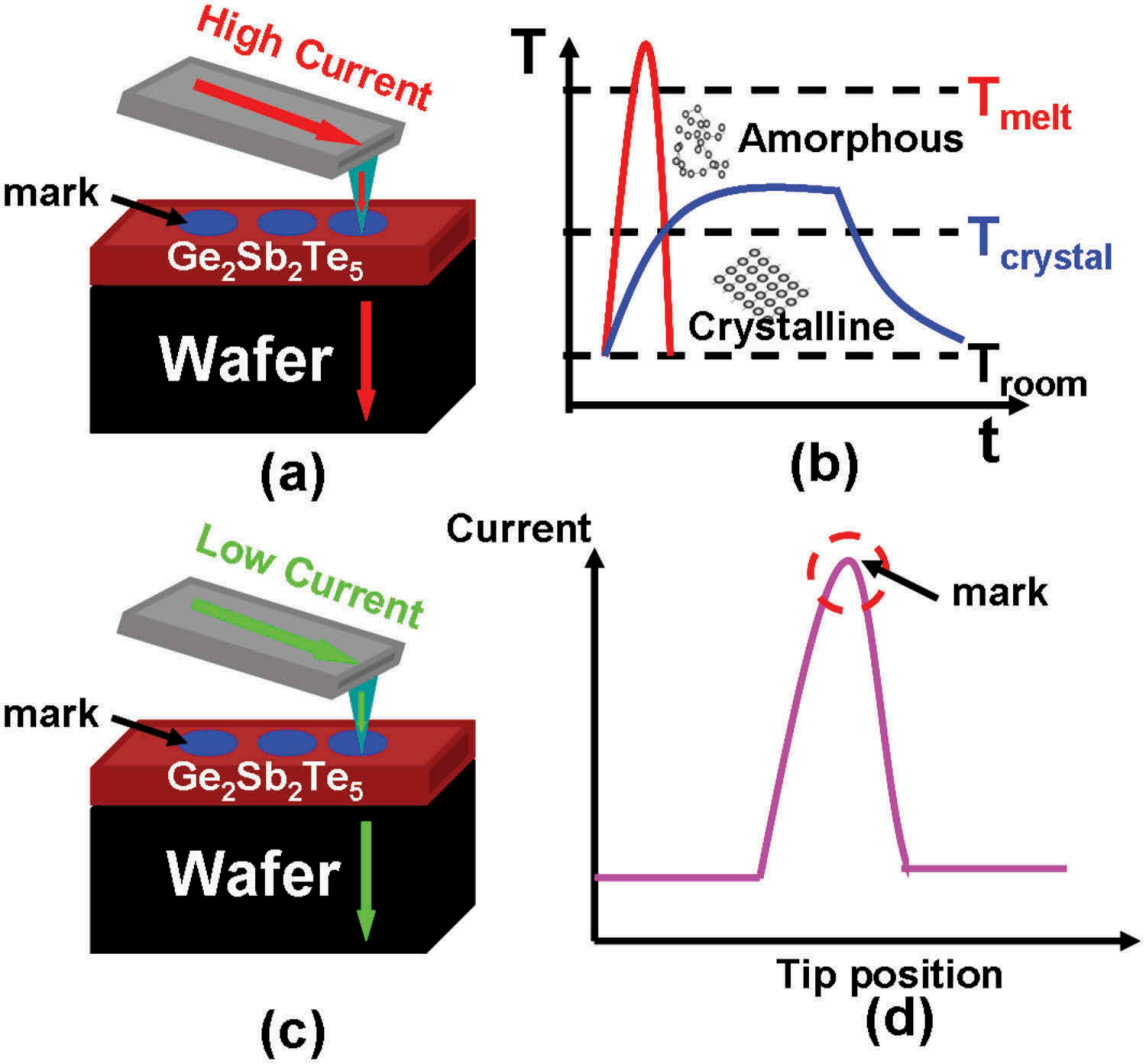
Electrical probe memory operated in (a) write mode and (c) readout mode, and the corresponding physical principle for (b) write process and (d) readout process.

Electrical probe memory using phase-change materials has been extensively considered as one of the most promising candidates for next-generation mass storage devices due to its potential for ultrahigh areal density, fast switching speed, low energy consumption, and long data retention time, arising from the inherent characteristics of nanoscale probe tip and phase-change storage media [1,2]. Recording is realized by injecting an electric pulse, via a nanoscale conductive probe, into the phase-change media (e.g. Ge_2_Sb_2_Te_5_) to either heat amorphous materials to its crystalline temperature to form crystalline mark or to heat crystalline materials to its molten phase followed by a rapid quench to generate amorphous mark, while readout is accomplished by applying a low voltage potential to the phase-change media and subsequently sensing the resulting current difference from the vast resistivity variation between amorphous and crystalline states, as illustrated in Figure 1.

A typical electrical probe memory consists of a Ge_2_Sb_2_Te_5_ thin film sandwiched by a capping layer served to protect the active media from wear and oxidation, and a bottom layer that acts as an electrode to collect write/read current, all of which are deposited on the silicon (Si) wafer. It was revealed in previous literatures that a thin capping layer with an intermediate electrical conductivity and low thermal conductivity is preferred to maintain sufficient Joule heat inside the active layer, whereas the bottom layer generally demands for high electrical conductivity to enable large/readout current, and low thermal conductivity aiming to attenuate the heat dissipation through the Si wafer [3]. Accordingly, the diamond-like carbon (DLC) film and the titanium nitride (TiN) film have been widely adopted for the capping and bottom medium of the practical electrical probe memory, respectively.

Although the advantageous features of the DLC such as high mechanical hardness, mature deposition techniques, and a large range of reconfigurable electrothermal properties are already demonstrated [4], the prospect of using DLC film for the capping layer of electrical probe memory has been drastically challenged mainly due to the difficulty in experimentally achieving the theoretically optimized properties (e.g. thickness, electrical conductivity, and thermal conductivity) [5]. Additionally, the electrical conductivity of the DLC capping layer was previously optimized to be 50–100 Ω^−1^ m^−1^ and therefore causes a large contact resistance at the tip–capping interface [6] that can be approximated by

(1) $$R_{contact}=\frac{\rho_{capping}\;\;+\;\;\rho_{tip}}{4{\left\{r^{2}-{\left[r-{\left(\frac{9F^{2}}{16r{E^{*}}^{2}}\right)}^{1/3}\right]}^{2}\right\}}^{1/2}},$$

where *ρ*_capping_ and *ρ*_tip_ are the resistivities of the capping layer and tip, respectively; *r* is the radius of tip apex; *F* is the tip loading force; and E* is the effective Young’s modulus. Such a high interfacial resistance indeed requires a higher electric stimulus for either crystallization or amorphization. Thanks to this, the practicality of utilizing TiN film for both capping and bottom layers has most recently been subjected to some preliminary investigation [7]. Because of its super-high electrical conductivity, the TiN capping layer allows for much lower contact resistance than that with the DLC capping layer, and no evidence of a reaction between TiN and Ge_2_Sb_2_Te_5_ has been found to date. However, the fairly high thermal conductivity of the TiN film (12 W m^−1^ K^−1^) exacerbates the heat leakage via the Si probe/wafer, increasing the energy consumption for the required phase-transformation. More importantly, the Ti ions may readily diffuse into the Ge_2_Sb_2_Te_5_ layer during SET/RESET processes and degrade the device endurance [8]. Accordingly, the previous design of the electrical probe memory needs to be significantly improved, necessitating an exploration of a more appropriate material for a capping and bottom layer of the electrical probe memory.

## 2. Methods

Indium tin oxide (ITO) films have widespread applications such as architectural coatings and transparent conductive electrodes in liquid crystal displays and solar cells owing to their high electrical conductivity (up to 10^6^ Ω^−1^ m^−1^) [9,10] and high optical transmission in the visible range. Their thermal conductivity is approximately 4 W m^−1^ K^−1^ [11] and can be further reduced to 0.84 ± 0.12 W m^−1^ K^−1^ [12] when the film is grown by spray pyrolysis. As a result, the possibility of using ITO films for both a capping and bottom layer of the electrical probe memory becomes very attractive.

**Figure 2.**
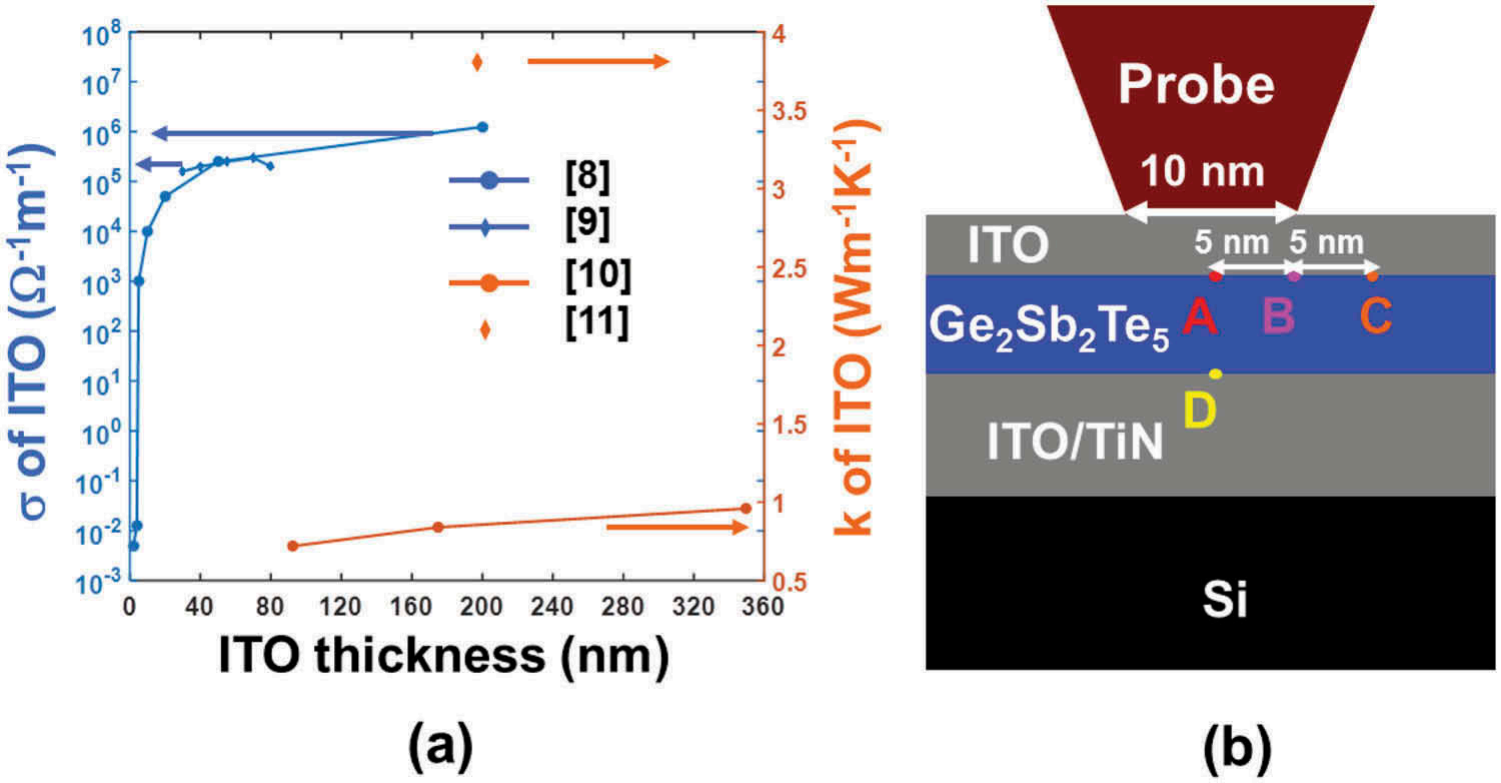
(a) The electrical (*σ*) and thermal (*k*) conductivity values of ITO films for different thickness and (b) several predefined points where phase-transformation temperature is calculated.

Figure 2(a) shows that the electrical and thermal conductivities of the ITO films may range from 5 × 10^−3^ to 1.25 × 10^6^ Ω^−1^ m^−1^ and from 0.84 ± 0.12 W m^−1^ K^−1^ to approximately 4 W m^−1^ K^−1^, respectively, when the thickness is varied from 2 to 200 nm and from 90 to 340 nm respectively. In this case, a previously developed three-dimensional (3D) numerical model [13,14] that coupled the Laplace equation, the classical heat conduction equation, and the rate equation is adopted to assess the impact of the ITO capping and bottom layer on the phase-transformation extent of the Ge_2_Sb_2_Te_5_ layer inside the electrical probe memory, defined by

(2) ∇⋅(σ⋅∇V)=0,

(3) $$\rho C_{p}\frac{\partial T}{\partial t}-k\cdot \nabla^{2}T=\sigma {\left|E\right|}^{2},$$

(4) $$\frac{\partial \chi }{\partial t}=A_{c}(1-\chi {)}^{n}\exp(\frac{-E_{c}}{k_{B}T}),$$

where *V* is the electric potential, *σ* is the electrical conductivity, *ρ* is the density, *C*_p_ is the heat capacity, *T* is the temperature, *k* is the thermal conductivity, *E* is the electric field, *χ* is the fraction of the crystal volume, *A*_c_ is the pre-factor, *n* is the reaction order, *E*_c_ is the height of the energy barrier for crystallization, and *k*_B_ is the Boltzmann’s constant. The Laplace equation is solved to provide the electrical field distribution inside the Ge_2_Sb_2_Te_5_ layer that is considered as the source of the Joule heating and is subsequently introduced to the heat conduction equation to calculate the consequent temperature; the resulting temperature is then implemented to supply with either the amorphization extent or the crystallization degree with the help of the rate equation. It should be noted that the electrical/thermal conductivities of the Ge_2_Sb_2_Te_5_ layer in this model pertain to electric field, temperature, and the resulting phase change [15,16], which were described elsewhere [14] and thus not repeated here. According to this model, the influence of the ITO thin film on the write performances of the electrical probe memory is reflected by calculating the maximum/minimum temperature at four predefined points inside the Ge_2_Sb_2_Te_5_ layer, as illustrated in Figure 2(b). To meet the ultrahigh recording density up to 10 Tbit/in^2^, the minimum temperature at points A (at the interface between the capping layer and Ge_2_Sb_2_Te_5_ layer and directly underneath the tip), point B (at the interface between the capping layer and Ge_2_Sb_2_Te_5_ layer and underneath the tip edge), and point D (at the interface between the bottom layer and Ge_2_Sb_2_Te_5_ layer and directly underneath the tip) is required to achieve either 400 °C for crystallization or 620 °C for amorphization, while the maximum temperature at point C (5 nm away from point B to ensure 10 Tbit/in^2^) needs to be less than 400 °C for amorphization and 620 °C for crystallization respectively to avoid the adverse impact of the thermal cross-talk effect on the previously recorded bit. Besides this, the maximum temperature at point A can not exceed 1400 °C so as to maintain the thermal stability of the ITO thin film [17].

To accurately imitate the practical environment, an electric excitation is applied to the top boundary of the tip during simulations, whereas the bottom boundary of the ITO bottom layer is ground. These two boundaries are also remained at room temperature, while other boundaries are set to be electrically or thermally insulated. The aforementioned model was solved numerically using a commercial software package – COMSOL MULTIPHYSICS^TM^ – based on finite-element method.

**Figure 3.**
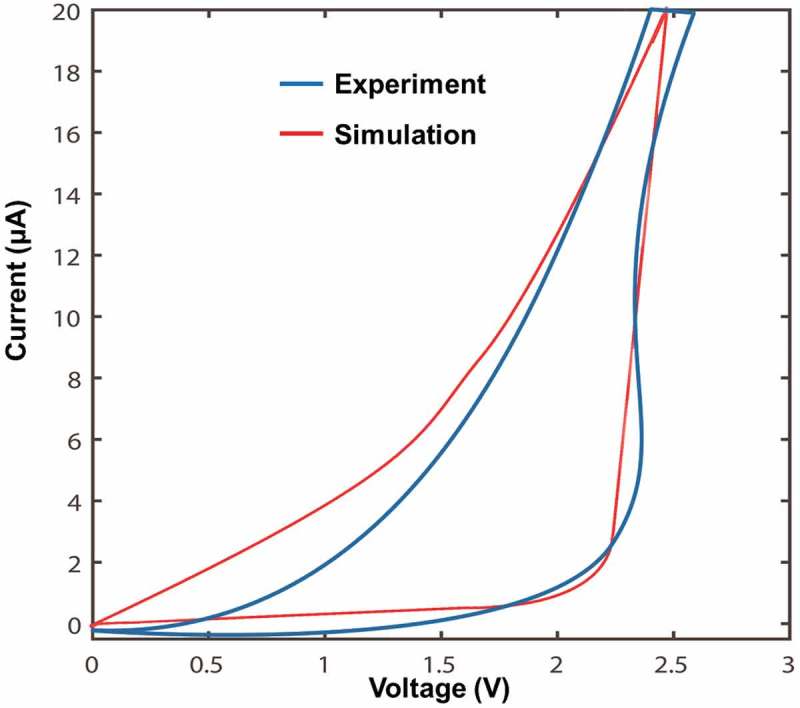
Comparison between an experimental I–V curve from a typical cell consisting of a 7-nm amorphous Ge_2_Sb_2_Te_5_ layer sandwiched by a 10-nm ITO top layer and a 50-nm ITO bottom layer and that calculated based on our theoretical model. The experimental I–V curve is replotted from Ref. [18].

As device optimization is performed by the developed model involving electrothermal and phase-transformation processes, its physical reality needs to be demonstrated prior to the detailed analysis. In this case, we compared an experimentally measured current–voltage (I–V) curve [18] from an ITO/Ge_2_Sb_2_Te_5_/ITO cell with that calculated using our theoretical model, as illustrated in Figure 3. Both experimental and calculated I–V curves clearly reveal that the resulting current initially remains extremely low when the applied voltage across the Ge_2_Sb_2_Te_5_ layer is below the so-called threshold value, while it suddenly undergoes a drastic increase once the threshold voltage is reached. Although the calculated I–V curve does not completely coincide with the experimental curve probably due to the uncertainty as to the ‘correct’ values of the chosen physical parameters, our theoretical model enables a similar current magnitude and a close threshold voltage to the experimental case, meaning that it can accurately mimic the threshold behavior of the Ge_2_Sb_2_Te_5_ media and can be therefore employed for the device optimization without having any physical ambiguity.

## 3. Results

### 3.1 Optimization of electrical conductivity and thickness of capping layer

Based on above scenario, the maximum and minimum temperatures at points A–D are first computed by varying the electrical conductivity and the thickness of the ITO capping film from 5 × 10^−3^ to 1.25 × 10^6^ Ω^−1^ m^−1^ and from 2 to 10 nm to evaluate the role of the ITO capping film on the phase-transformation process of the electrical probe memory. Note that the ITO capping films with a thickness >10 nm are not taken into account here due to their largely inherent resistance that causes much higher energy consumption, and the write excitations are set to be a 4-V pulse of 200 ns (50 ns rising, 100 ns plateau, and 50 ns trailing edges) for crystallization and a 5-V pulse of 200 ns (10 ns rising [time taken to change from 0 to the maximum magnitude], 180 ns plateau, and 10 ns trailing edges) for amorphization, respectively. A steeper pulse for amorphization is adopted here so as to maintain sufficiently high cooling rate. Note that for each configuration, the contact resistance at tip–capping interface that is calculated separately as a function of the interfacial mechanical/electronic properties (e.g. applied force, effective Young’s modulus, effective Poisson ratio, and electrical resistivities of tip and ITO capping) [19] is introduced into 3D simulations to closely mimic the interfacial conditions. All the characteristic modeling parameters are given in Table 1.

**Table 1.** Characteristic parameters used in simulations.

	Tip	ITO capping	Ge_2_Sb_2_Te_5_	ITO bottom	Si
Thickness (nm)	10	2–10	10	2–200	1000
Density (kg m^−3^)	12,400	7120	6150	7120	2330
Heat capacity (J kg^−1^ m^−1^)	250	341	210	341	720
Thermal conductivity (W m^−1^ K^−1^)	25	0.84/4	0.2/0.58	0.84/4	149
Electrical conductivity (Ω^−1^ m^−1^)	3.3 × 10^6^	5 × 10^−3^–1.25 × 10^6^	E/T dependent	5 × 10^−3^–1.25 × 10^6^	N/A
Tip force (nN)	300	N/A	N/A	N/A	N/A
Young’s modulus (GPa)	159	116	N/A	N/A	N/A
Poisson ratio	0.32	0.35	N/A	N/A	N/A

**Figure 4.**
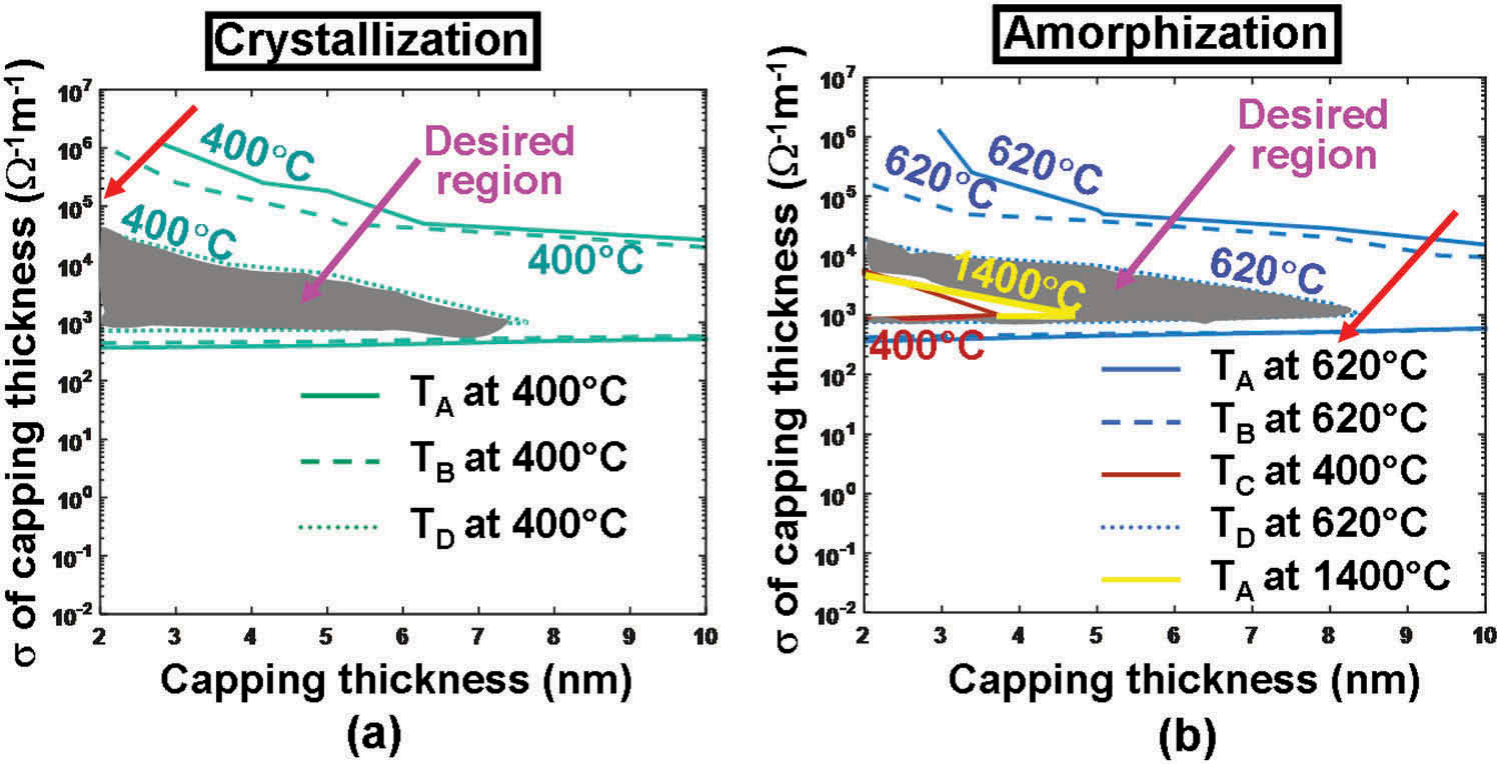
Temperature contours at points A, B, C, D as a function of electrical conductivity (*σ*) and thickness of the ITO capping layer during (a) crystallization and (b) amorphization processes. For both simulations, the thermal conductivity of the capping layer remains at 0.84 W m^−1^ K^−1^, while the bottom layer is assumed to be TiN layer with a thickness, an electrical conductivity, and a thermal conductivity of 40 nm, 10^6^ Ω^−1^ m^−1^, and 12 W m^−1^ K^−1^, respectively. Note that for (a) maximum temperature contours of 1400 °C at A and 400 °C at C are outside this figure and, therefore, are not visible. The red arrow indicates the direction along which the temperature increases.

Figure 4(a) reveals that for a given electrical conductivity, the use of a thicker ITO capping layer would remarkably reduce the temperature at these probe points, clearly matching previous observations. This can be readily understood since a thicker ITO capping creates a more electrically resistive path between the tip and the active layer and thereby results in lower joule heating than that with thinner ITO capping. Another crucial finding from Figure 4(a) is that increasing the electrical conductivity of the ITO capping layer with a fixed thickness turns out to decrease the temperature at points A–D, converse to previous reports elucidating that the temperature inside the Ge_2_Sb_2_Te_5_ layer can be heavily enhanced by increasing the electrical conductivity of the DLC capping [20]. This discrepancy can be attributed to the large difference on the investigated electrical conductivity between ITO capping that varies from 5 × 10^−3^ to 1.25 × 10^6^ Ω^−1^ m^−1^ and DLC capping from 10 to 100 Ω^−1^ m^−1^. DLC capping exhibits a similar electrical conductivity to that of the amorphous Ge_2_Sb_2_Te_5_ and consequently the write current prefers to flow vertically through the region underneath the tip, whereas for ITO capping with an electrical conductivity much higher than the amorphous Ge_2_Sb_2_Te_5_, majority of current would spread along the ITO capping itself other than penetrating through the Ge_2_Sb_2_Te_5_ media and therefore gives rise to lower temperature for higher electrical conductivity. As a result, the optimum values of the electrical conductivity and the thickness of the ITO capping to meet the aforementioned crystallization temperature requirements for points A–D range from 2 to 7 nm and from 10^3^ to 0.5 × 10^5^ Ω^−1^ m^−1^, respectively, constrained in the gray region shown in Figure 4(a). Notably, the 620 and 1400 °C contours are outside the plot, meaning that they only occur for voltage >4 V.

The dependences of the temperature at probe points on the thickness and electrical conductivity of the ITO capping are similar for amorphization and crystallization processes, as illustrated in Figure 4(b). The assessed temperature can be increased by either reducing the thickness or increasing the electrical conductivity of the ITO capping layer with the reasons elaborated above. Therefore, the optimum values of the electrical conductivity and the thickness of the ITO capping layer for amorphization are chosen in such a way that the maximum temperature at points A, B, and D needs to reach at least 620 °C, while the maximum temperature at points A and C can not exceed 1400 °C and 400 °C, respectively. Hence, the configurations whose electrical conductivity varies from 10^3^ to 10^4^ Ω^−1^ m^−1^ and thickness from 4.5 to 8.5 nm, located in the gray region illustrated in Figure 4(b), seem to fulfill the requirements. As a result, in order to satisfy temperature requirements for both crystallization and amorphization, the appropriate electrical conductivity and the thickness of the ITO capping are found between 10^3^ to 10^4^ Ω^−1^ m^−1^, and between 4.5 and 7 nm, respectively. Considering the experimental availability of the film structural/electronic properties shown in Figure 2(a), the thickness and the electrical conductivity of the ITO capping layer are optimized here to be 5 nm and 10^3^ Ω^−1^ m^−1^, respectively.

**Figure 5.**
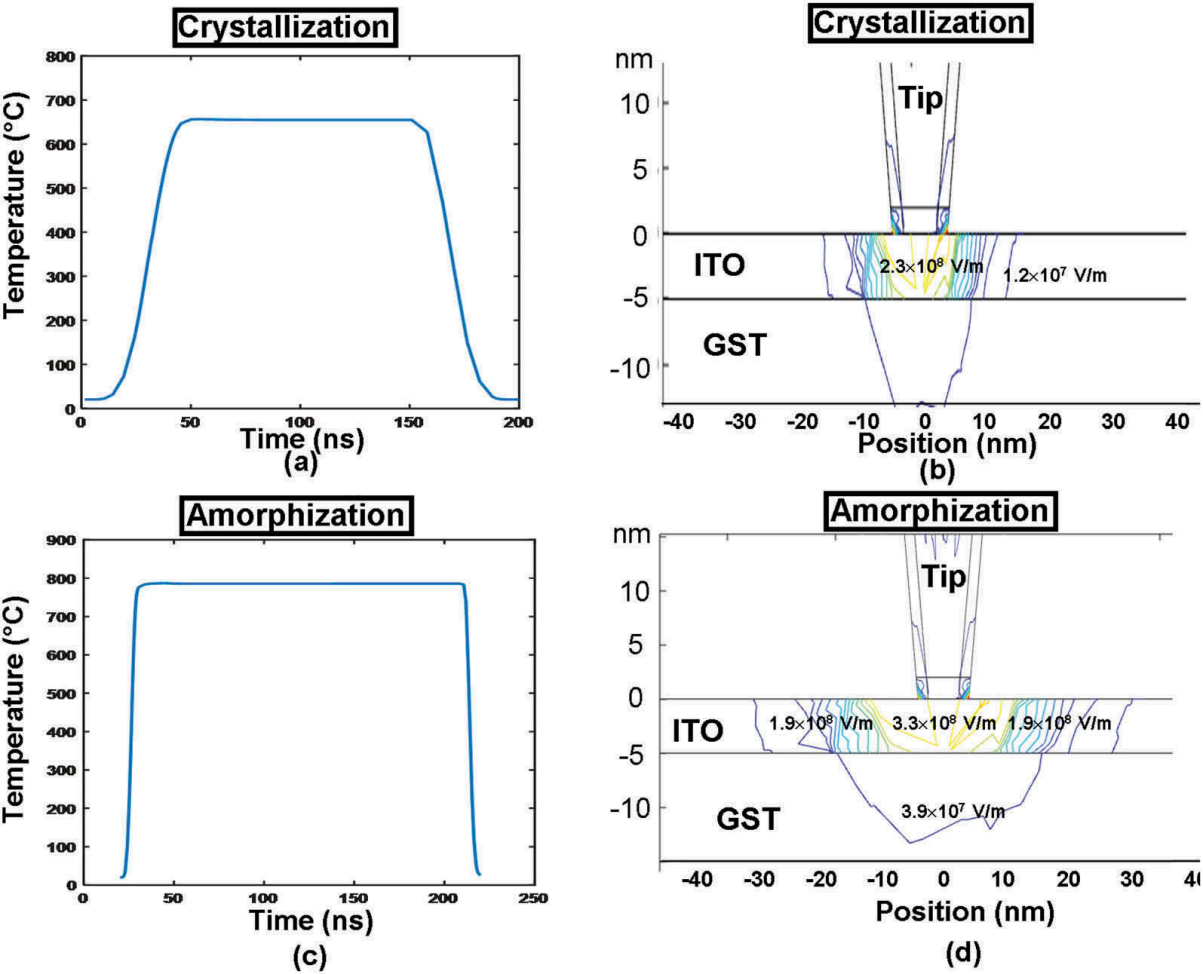
The temperature variation in the middle of the ITO capping (directly underneath the tip) as a function of the pulse time for (a) crystallization and (b) amorphization, and the electric field distribution inside the ITO capping at the onset of the pulse plateau for (c) crystallization and (d) amorphization. The electrical conductivity, thickness, and thermal conductivity of the capping layer are set to be 10^3^ Ω^−1^ m^−1^, 5 nm, and 0.84 W m^−1^ K^−1^, while other parameters are as for Figure 4.

To further understand the role of ITO capping layer on the write performances of the electrical probe memory, the change of the temperature in the middle of the ITO capping as a function of the pulse time and the electrical field distribution inside the ITO capping at the onset of pulse plateau were calculated for both crystallization and amorphization processes, giving rise to Figure 5.

It is revealed in Figure 5(a,c) that the temperature change inside the ITO capping almost matches the trend of the pulse variation, as it reaches the maximum temperature at the end of the pulse rising edge, and remains constant during the pulse plateau, subsequently falling back to room temperature at the end of the pulse trailing edge. This obviously allows for a sufficiently high heating rate inside the Ge_2_Sb_2_Te_5_ layer where the required phase-transformation temperature and the satisfying cooling rate for both crystallization and amorphization can be readily achieved (particularly useful for amorphization due to its short rising edge). Further observations also indicate that the maximum temperature reached inside the ITO capping was found to be 600 °C for crystallization and 800 °C for amorphization, respectively, far below its high temperature limit (1400 °C) and thus ensuring the device reliability. Besides, the electrical fields inside the ITO capping, according to Figure 5(b,d), are found to be uniform through the whole ITO thickness due to its large electrical conductivity, which would ensure high electrical field/current density generated inside the Ge_2_Sb_2_Te_5_ layer to lead to adequate joule heating for the required phase transformation. Note that the electric field distribution shown in Figure 5 does not strictly follow a radial symmetry probably due to the non-uniform mesh distribution underneath the tip.

### 3.2 Optimization of thermal conductivity and thickness of capping layer

**Figure 6.**
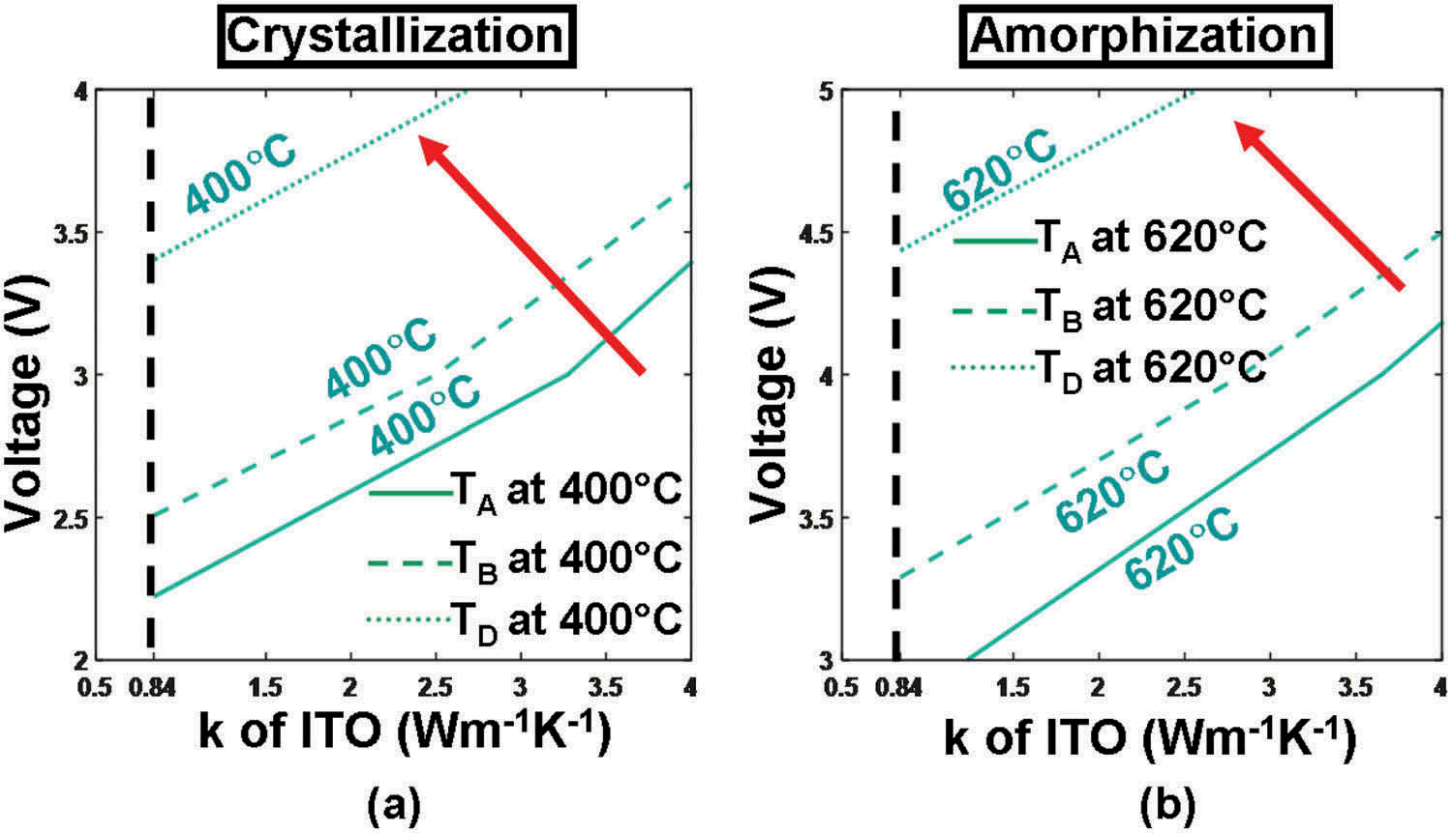
Temperature contours at points A, B, C, D as a function of thermal conductivity (*k*) and thickness of the ITO capping layer during (a) crystallization and (b) amorphization processes. For both simulations, the thickness and the electrical conductivity of the capping layer remain at 5 nm and 10^3^ Ω^−1^ m^−1^, while other parameters are as for Figure 3. Note that for both figures, maximum temperature contours of 1400 °C at A and 400 °C at C are outside the range and, therefore, are not visible. The red arrow indicates the direction along which the temperature increases.

According to literature [21,22], thermal conductivity of the capping layer that determines the conductive path of joule heating also plays a critical role in determining the maximum/minimum temperature inside the active layer, thus requiring particular attention. Hence, the maximum/minimum temperature at points A–D is reevaluated by changing the thermal conductivity of the ITO layer (i.e. 0.84 and 4 W m^−1^ K^−1^) for various pulse magnitudes (2–4 V for crystallization and 3–5 V for amorphization), as depicted in Figure 6. It is clearly indicated in Figure 6 that utilizing a higher pulse magnitude or an ITO capping with a lower thermal conductivity can result in higher temperature at detected points for both crystallization and amorphization processes. Obviously a large write pulse can readily give rise to more joule heating at the expense of the extra power/energy consumption. The use of an ITO capping with low thermal conductivity would effectively prevent joule heating from dissipating through the probe tip and make the active media heated sufficiently. According to Figure 6, neither of the required temperatures (i.e. 400 °C for crystallization and 620 °C for amorphization) can be achieved using an ITO capping with a thermal conductivity of 4 W m^−1^ K^−1^, implying that the thermal conductivity of the ITO capping needs to be ~0.84 W m^−1^ K^−1^. As also revealed from Figure 6, the required temperature can be secured by further reducing the pulse magnitude to 3.3 V for crystallization and 4.3 V for amorphization, much smaller than the cases with DLC capping and thereby allowing for lower power/energy consumption.

### 3.3 Optimization of electrical conductivity and thickness of bottom layer

**Figure 7.**
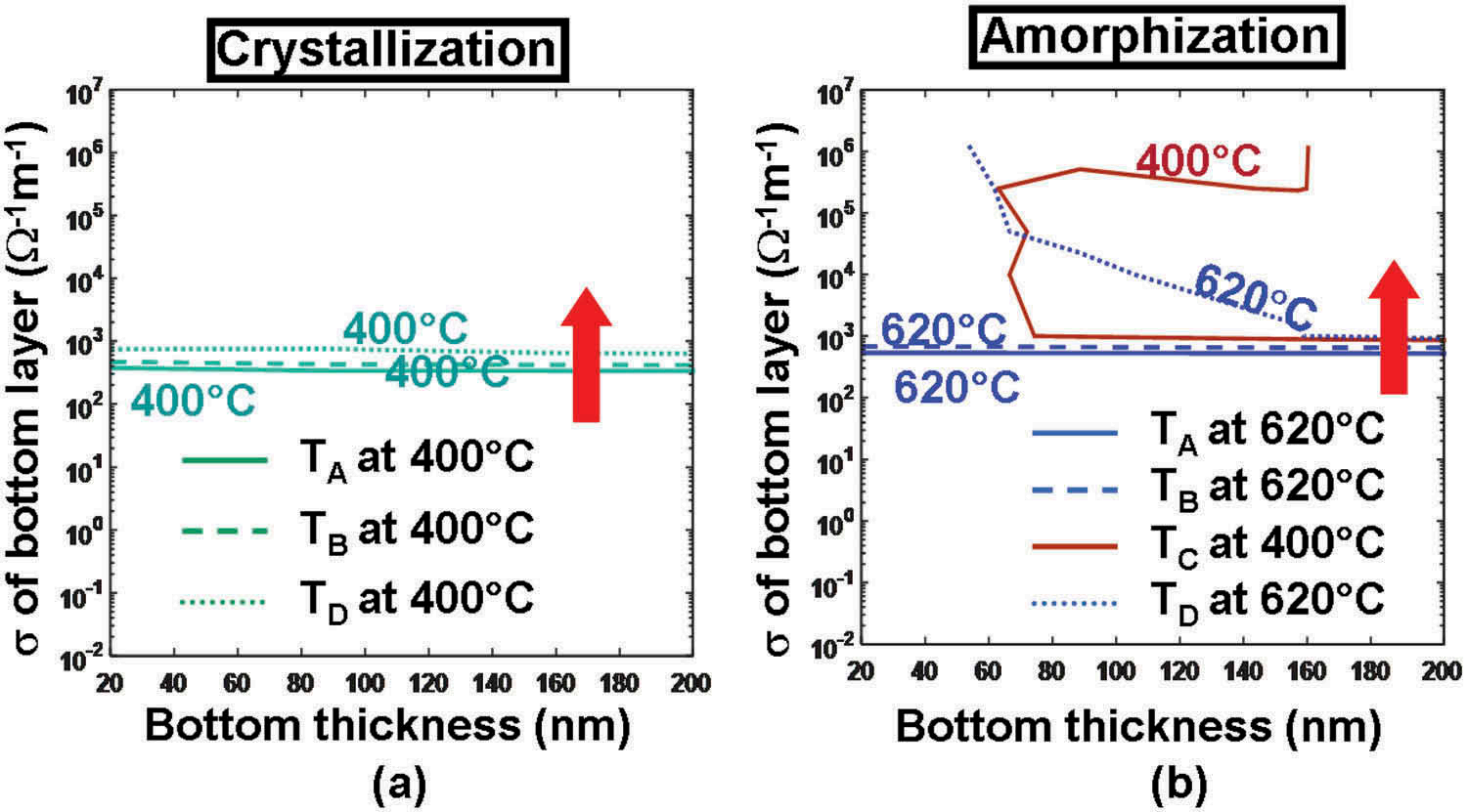
Temperature contours at points A, B, C, D as a function of electrical conductivity (*σ*) and thickness of the ITO bottom layer during (a) crystallization and (b) amorphization processes. For both simulations, the thickness, the electrical conductivity, and the thermal conductivity of the capping layer remain at 5 nm, 10^3^ Ω^−1^ m^−1^, and 0.84 Wm^−1^ K^−1^, while the thermal conductivity of the ITO bottom layer remains at 0.84 Wm^−1^ K^−1^. Note that for (a) maximum temperature contours of 1400 °C at A and 400 °C at C are outside the range and, therefore, are not visible. The red arrow indicates the direction along which the temperature increases.

The bottom layer of the electrical probe memory is deployed to collect the write/read current and may have a strong impact on the resulting current density as well as the temperature. To establish their pertinence, the previous TiN bottom layer is replaced by an ITO film whose thickness and electrical conductivity vary from 2 to 200 nm and from 10^3^ to 1.25 × 10^6^ Ω^−1^ m^−1^, respectively, and the resulting maximum/minimum temperature at points A–D is recalculated according to a crystallization pulse of 3.3 V of 200 ns and an amorphization pulse of 4.3 V of 200 ns, leading to Figure 7.

According to Figure 7, the use of either a thicker ITO bottom for a fixed electrical conductivity or a more electrically conductive ITO bottom with a fixed thickness can slightly increase the temperature inside the active layer for both crystallization and amorphization processes. It is evident that an ITO bottom with large electrical conductivity can somewhat reduce the whole device resistance and is therefore capable of generating more joule heating. Additionally, a thicker ITO bottom layer can further separate the active layer from the Si substrate that is widely considered as a heat sink, guaranteeing that the bottom of the active layer can be heated adequately. It is clearly indicated in Figure 7(a) that the required temperature for crystallization at points A–D can be met with an electrical conductivity and a thickness of the ITO bottom greater than 0.5 × 10^2^ Ω^−1^ m^−1^ and 20 nm, respectively, while in order to satisfy the amorphization temperature, the practicable thickness and electrical conductivity lie in the ranges from 70 to 200 nm and from 10^5^ to 1.25 × 10^6^ Ω^−1^ m^−1^, respectively. As a result, the electrical conductivity and the thickness of the ITO bottom layer chosen to fulfill the temperature requirements for both crystallization and amorphization should vary from 10^5^ to 1.25 × 10^6^ Ω^−1^ m^−1^ and from 70 to 200 nm, respectively. To account for the state of the art in ITO film deposition and to suppress the heat dissipation through the Si substrate, the electrical conductivity and the thickness of the ITO bottom layer are optimized to be 1.25 × 10^6^ Ω^−1^m^−1^ and 200 nm, respectively.

### 3.4 Optimization of thermal conductivity and thickness of bottom layer

**Figure 8.**
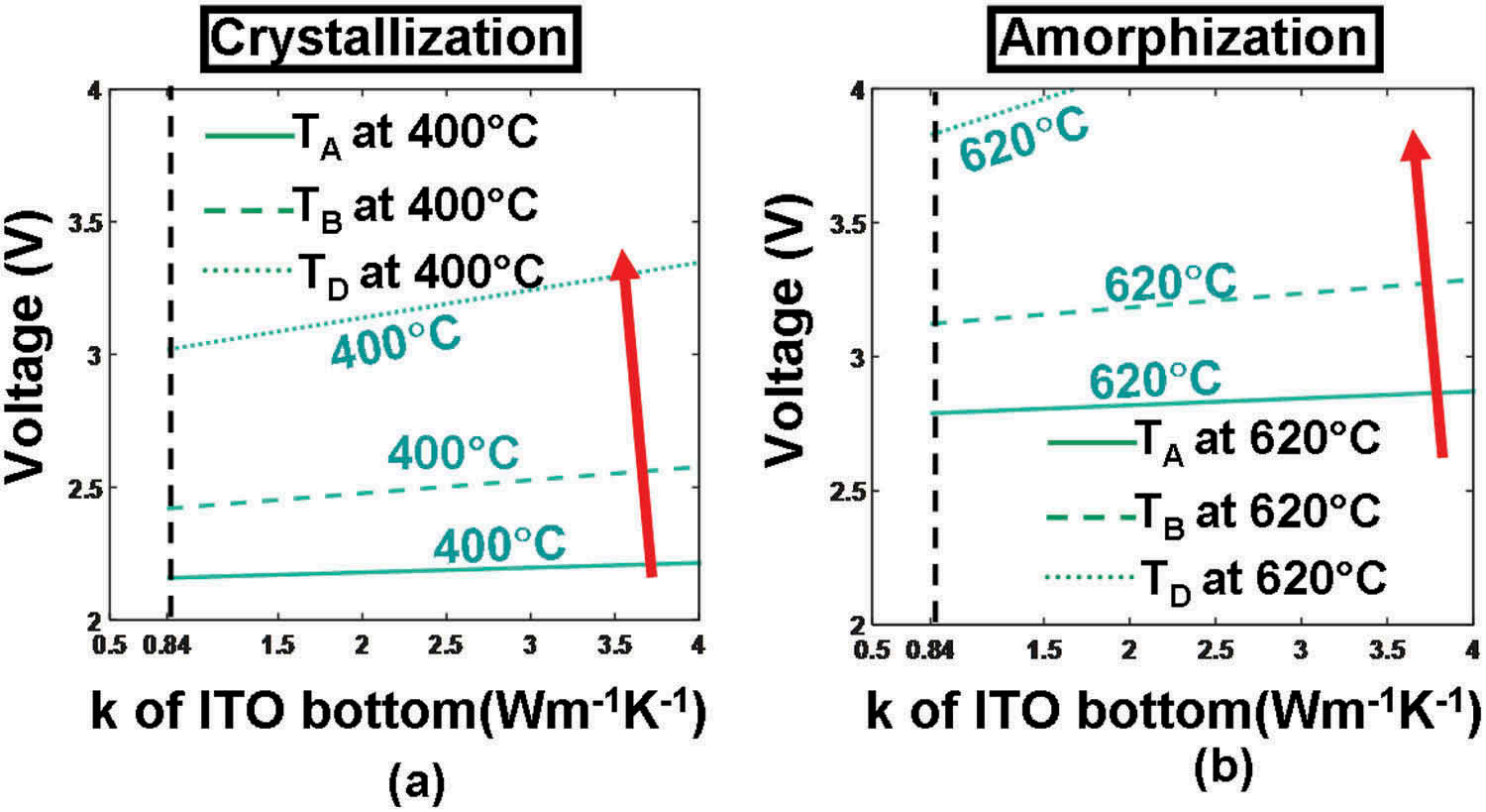
Temperature contours at points A, B, C, D as a function of thermal conductivity (*k*) and thickness of the ITO bottom layer during (a) crystallization and (b) amorphization processes. For both simulations, the thickness, the electrical conductivity, and the thermal conductivity of the capping layer remain at 5 nm, 10^3^ Ω^−1^ m^−1^, 0.84 W m^−1^ K^−1^, while other parameters are as for Figure 5. Note that for both figures, maximum temperature contours of 1400 °C at A and 400 °C at C are outside the range and, therefore, are not visible. The red arrow indicates the direction along which the temperature of these points increases.

In addition to its electrical conductivity and thickness, the thermal conductivity of the bottom layer determines the extent to heat dissipation toward the Si substrate and may thus have a pronounced impact on the temperature at probe points, particularly for D. Similar to the capping layer case, the temperature at points A–D here is recalculated for different pulses by varying the thermal conductivity of the ITO bottom layer (i.e. 0.84 and 4 W m^−1^ K^−1^), resulting in Figure 8. It reveals that the use of an ITO bottom layer with a larger thermal conductivity (4 W m^−1^ K^−1^ in this case) would allow more joule heating to escape from the bottom substrate and consequently give rise to lower temperature at points A–D than its counterpart with smaller thermal conductivity (i.e. 0.84 W m^−1^ K^−1^) for both crystallization and amorphization processes. Another intriguing observation from Figure 8 is that using an ITO bottom layer with a thermal conductivity of either 0.84 or 4 W m^−1^ K^−1^ can successfully accomplish crystallization within the investigated voltage scope (i.e. 2–4 V), while amorphization can be realized by an ITO bottom layer with a thermal conductivity of 0.84 W m^−1^ K^−1^ only within the same voltage range. In this case, the thermal conductivity of the ITO bottom layer is optimized to be 0.84 W m^−1^ K^−1^, and the optimized voltage pulses for crystallization and amorphization are further decreased to 3 and 3.8 V, respectively.

## 4. Discussion

**Figure 9.**
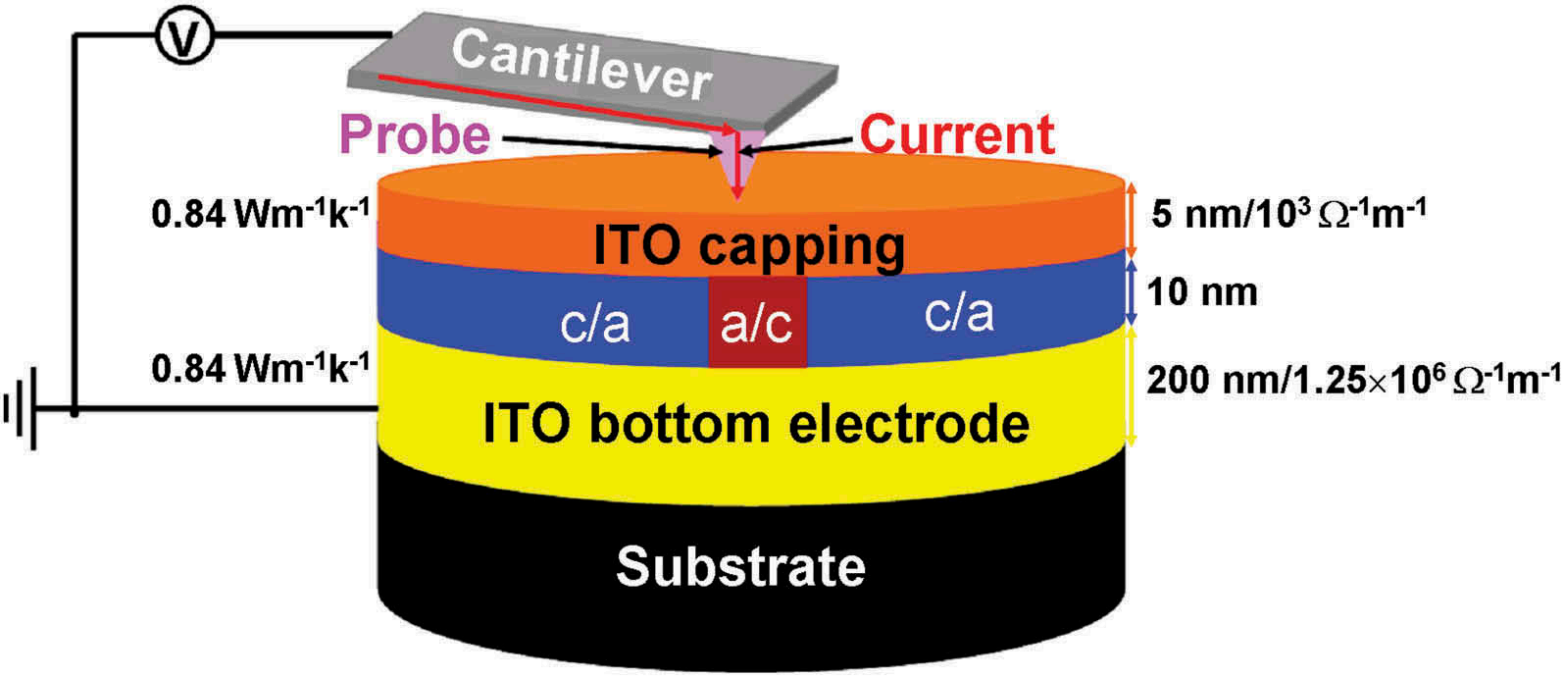
Schematic of the optimized electrical probe memory with ITO capping and bottom layers. ‘c’ and ‘a’ represent crystalline and amorphous phases, respectively.

Based on aforementioned analysis, the optimized architecture of the electrical probe memory is redesigned to comprise a SiO_2_ encapsulated Si tip with PtSi at the tip apex [23] and recorded tri-layer stack that consists of a 10-nm Ge_2_Sb_2_Te_5_ layer sandwiched by a 5-nm ITO capping layer with an electrical conductivity of 10^3^ Ω^−1^ m^−1^ and a thermal conductivity of 0.84 W m^−1^ K^−1^, and a 200-nm ITO bottom layer with an electrical conductivity of 1.25 × 10^6^ Ω^−1^ m^−1^ and a thermal conductivity of 0.84 W m^−1^ K^−1^, deposited on a Si wafer. Such a structure is schematically shown in Figure 9.

**Figure 10.**
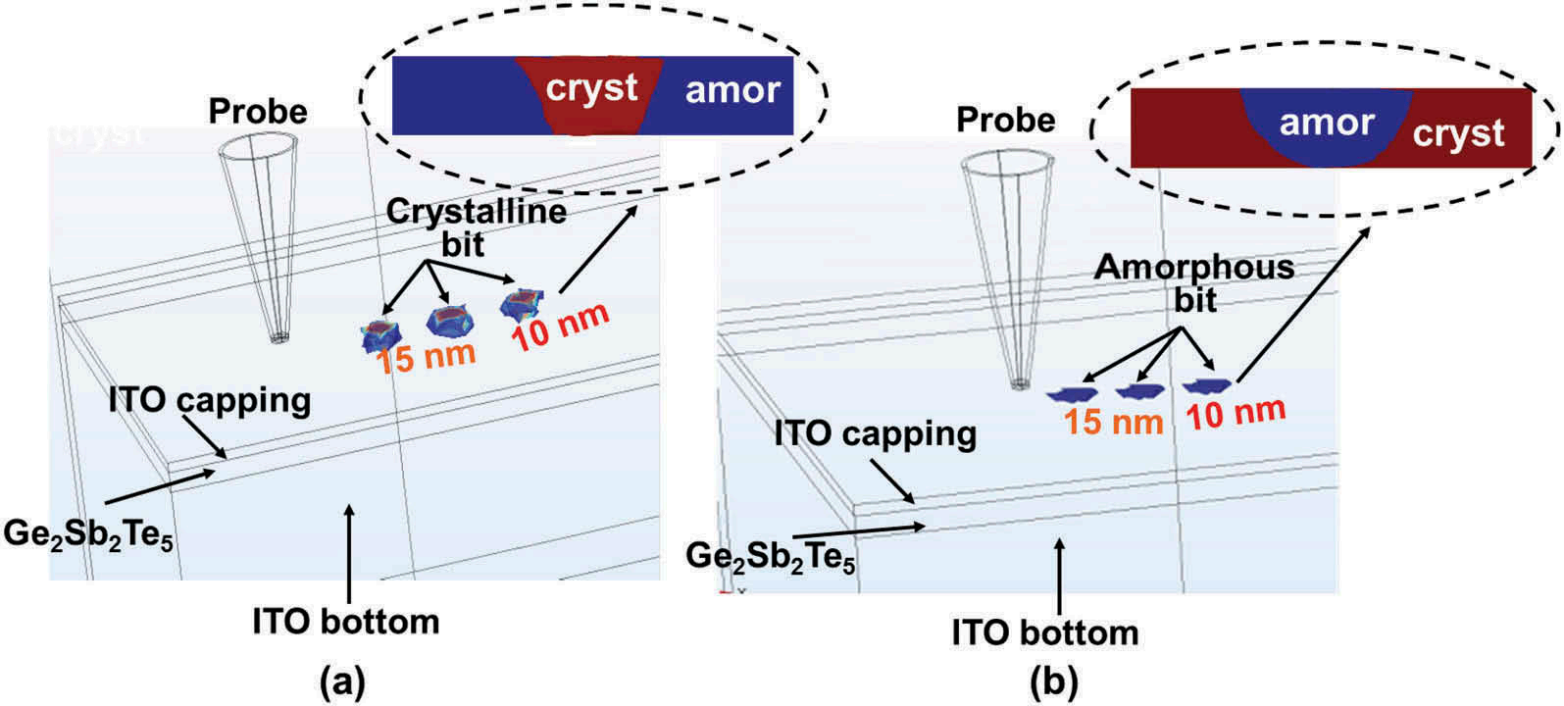
The resultant bit array in (a) crystalline and (b) amorphous phase from the optimized probe device with ITO capping and bottom layers. The insets show the cross-sectional view of the formed bits.

To assess its physical performances, the aforementioned 3D model was implemented again to mimic the electrothermal and phase-transformation kinetics occurring during the crystallization and amorphization of the Ge_2_Sb_2_Te_5_ layer inside this newly designed device with ITO capping and bottom layers, leading to Figure 10. It should be noted that such a model enables the formation of bit arrays and thereby allows for the investigation of thermal cross-talk effect between adjacent written bits, which can not be achieved by any 2D or pseudo-3D computational models reported previously [6,20]. As shown in Figure 10(a), a 1 × 3 crystalline bit array was produced with a 15-nm separation between two adjacent bit centers. It was found that the resulting crystalline bit exhibits a truncated-cone shape with a diameter of ~10 nm, extending through the whole Ge_2_Sb_2_Te_5_ layer. This evidently demonstrates the possibility of achieving multi-terabit/in^2^ areal density and discernible readout signal based on the electrical probe device with ITO capping and bottom layers [24]. On the other hand, the feasibility of implementing this device to generate a 1 × 3 amorphous bit array is also proven according to Figure 10(b). In contrast to the crystalline bit, the shape of a single amorphous bit that extends through the whole active layer is considered as semi-elliptical with a diameter of ~10 nm, also suggesting an ultrahigh areal density and a noticeable readout signal.

**Figure 11.**
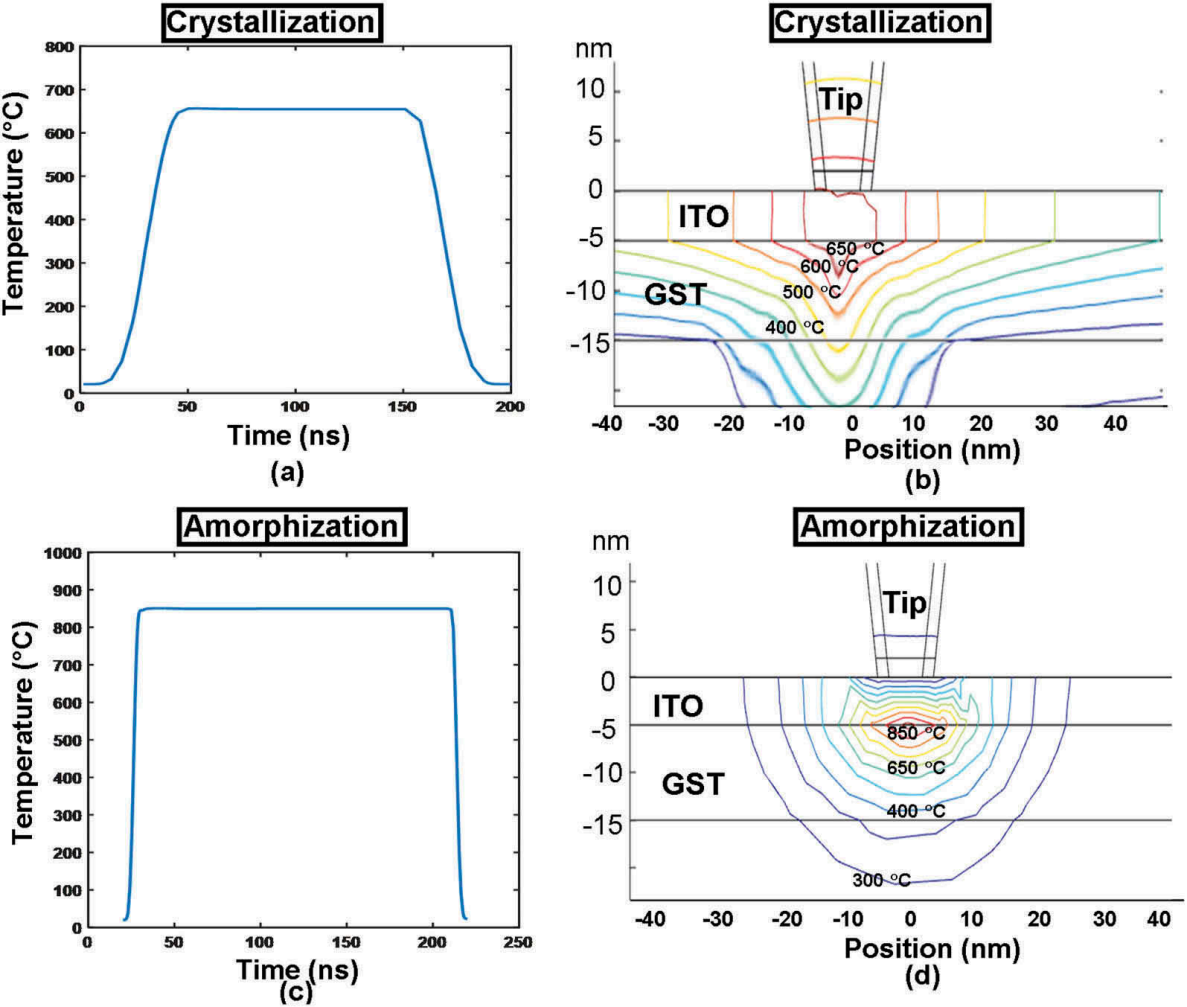
Temperature variation at point A as a function of the pulse time for (a) crystallization and (c) amorphization, and the temperature distribution at point A at the onset of the pulse plateau for (d) crystallization and (d) amorphization. The modeling parameters are as for Figure 10.

Such shape difference between resulting crystalline and amorphous bits may be ascribed to their respective temperature distribution inside the Ge_2_Sb_2_Te_5_ layer, as illustrated in Figure 11. As clearly shown in Figure 11(b,d), the temperature contour inside the active layer during crystallization process exhibits a quasi-triangular shape approximately, while the contour for the amorphization case presents an oval shape. Therefore, the size of the resulting crystalline and amorphous bits is intimately related to the regions encompassed by a temperature contour of 400 and 620 °C, respectively, thereby giving rise to a truncated-cone shaped crystalline bit and a semi-elliptical amorphous bit. Findings from Figure 11(a,c) also imply that using ITO capping and bottom layers can effectively raise the temperature inside the Ge_2_Sb_2_Te_5_ layer to the required values within the given pulse width, while avoiding excessive temperature inside the device. Additionally, the thermal cross-talk effect between two adjacent bits in either crystalline or amorphous phases is significantly eliminated even if the adjacent bit gap is reduced to 15 nm, clearly ensuring the thermal reliability of this newly designed device. More importantly, owing to the advantageous traits of applied pulses such as low magnitude and short width, the required write energy per bit was excitingly reduced to 0.02 pJ for a crystallization pulse of 3 V of 200 ns and 0.05 pJ for an amorphization pulse of 3.8 V of 200 ns, respectively. Furthermore, the contact resistance introduced at the tip-ITO capping interface is calculated to be ~8 kΩ with mechanical properties introduced in Table 1, which is much lower than the case of DLC capping [25] having a contact resistance of ~80 kΩ, and can be ignored when compared with the whole device resistance usually in the range of hundreds of kΩ. These encouraging findings undoubtedly reveal the ability of this optimized electrical probe memory with ITO capping and bottom layer to provide ultrahigh areal density within fJ energy consumption during *hundreds of* ns period, certainly making it more competitive than electrical probe memories with either DLC or TiN capping and bottom layers and other probe memories utilizing different active materials from phase-change media.

## 5. Conclusions

Phase-transformation temperature was calculated here at some predefined points to testify the practicality of harnessing ITO capping and bottom layers for electrical probe memory application by tailoring the electrical/thermal conductivities and thickness of the ITO capping and bottom layers within the experimentally reported values. It was found that an electrical probe memory, consisting of a SiO_2_ encapsulated Si tip with PtSi at tip apex of 10 nm diameter and a 10-nm Ge_2_Sb_2_Te_5_ layer sandwiched by a 5-nm ITO capping layer with an electrical conductivity of 10^3^ Ω^−1^ m^−1^ and a thermal conductivity of 0.84 W m^−1^ K^−1^, and a 200-nm ITO bottom layer with an electrical conductivity of 1.25 × 10^6^ Ω^−1^ m^−1^ and a thermal conductivity of 0.84 W m^−1^ K^−1^, enables the required temperature at predefined points for both crystallization and amorphization, while matching the experimentally measured properties of the ITO media. The practicality of using aforementioned optimized device to achieve ultrahigh density (multi-terabit/in^2^), ultralow energy consumption (~fJ), ultrafast switching speed (~ns), low interfacial contact resistance, and high thermal reliability has also been demonstrated.
